# The prevalence and prognostic value of frailty screening measures in patients undergoing surgery for colorectal cancer: observations from a systematic review

**DOI:** 10.1186/s12877-022-02928-5

**Published:** 2022-03-29

**Authors:** Josh McGovern, Ross D. Dolan, Paul G. Horgan, Barry J. Laird, Donald C. McMillan

**Affiliations:** 1 Academic Unit of Surgery, School of Medicine, University of Glasgow, New Lister Building, Royal Infirmary, Glasgow, G31 2ER UK; 2 Institute of Genetics and Cancer, University of Edinburgh, Edinburgh, EH4 2XU UK

**Keywords:** Frailty, Colorectal cancer, Clinical outcomes

## Abstract

**Introduction:**

Frailty is a complex multifactorial syndrome characterised by a significant increase in vulnerability and worsened health outcomes. Despite a range of proposed frailty screening measures, the prevalence and prognostic value of frailty in patients undergoing surgery for colorectal cancer is not clear.

**Aim:**

The aim of this present review was to examine the use of commonly employed frailty screening measures in patients undergoing surgery for colorectal cancer.

**Methods:**

A systematic search of PubMed and Medline was carried out to identify studies reporting the use of frailty screening tools or measures in patients undergoing surgery for colorectal cancer. The screening measure used and prevalence of frailty within the population were recorded. Outcomes of interest were the incidence of post-operative complications, 30-day mortality and overall survival.

**Results:**

Of the 15 studies included (*n* = 97, 898 patients), 9 studies were retrospective and included patients aged 70 years or older (*n* = 96, 120 patients). 5 of 12 studies reported that frailty was independently associated with the incidence of post-operative complications. There was also evidence that frailty was independently associated with 30-day mortality (1 of 4 studies, *n* = 9, 252 patients) and long-term survival (2 of 3 studies, *n* = 1, 420 patients).

**Conclusions:**

Frailty was common in patients with colorectal cancer and the assessment of frailty may have prognostic value in patients undergoing surgery. However, the basis of the relationship between frailty and post-operative outcomes is not clear and merits further study.

## Introduction

Colorectal cancer (CRC) accounts for approximately 12% of new cancer cases diagnosed within the UK each year [1]. Approximately half of all colorectal cancer cases are in patients aged 75 years and over [1]. Furthermore, while age-specific incidence rates vary, the highest rates observed are in the 85 to 89 age group, for both males and females [1]. Advanced age is associated with recognised prognostic factors including co-morbidity [2], sarcopenia [3] and frailty [4]. Therefore, decisions on whether to embark on potentially curative treatment are often complex in older adults with CRC.

Frailty is a complex multifactorial syndrome, characterised by a clinically significant increase in vulnerability and worsened health outcomes [4]. Given the multi-domain character of frailty, with both physical and psychological components contributing to the condition, diagnosing frailty can be difficult for non-experienced clinicians. At present, Comprehensive Geriatric Assessment (CGA) is viewed as the gold standard for diagnosing frailty [5]. The National Institute of Health Consensus Development define CGA as a multidisciplinary evaluation in which the multiple problems of older persons are uncovered, described, explained [6]. This facilitates assessment of the need for enhanced services and the development of a co-ordinated care plan, tailored to the patients. Use of the CGA is advocated in older patients with cancer by the International Society of Geriatric Oncology [7]. Recent cohort studies have shown that older adults patients identified as frail using CGA had significantly increased risk of severe complications [8] and worsened survival outcomes after elective surgery for colorectal cancer [9]. However, CGA is time consuming, with benefit determined by inter-department collaborative care and frailty-targeted optimized intervention programs [10, 11].

In recent years a number of frailty screening measures have been developed to aid physicians in diagnosing frailty [12]. These range in modality, criteria assessed, objectivity and patient participation. Common examples in the current literature range from the simple, image-based Canadian Study of Health and Aging-Clinical Frailty Scale (CSHA-CFS) [13], to the American College of Surgeons National Surgical Quality Improvement Program (ACS NSQIP) Modified frailty indices [14, 15], which combine performance status and co-morbidity, to multi-modal screening measures which include assessments of functional and nutritional status, co-morbidity and subjective, patient-determined elements; examples include the Edmonton Frail Scale [16], Groningen Frailty Indicator [17], Onco-geriatric G8 questionnaire and frailty phenotype [18].

Despite the range of screening measures available, there is a paucity of research examining the prevalence of frailty and the prognostic value of these measures, in patients undergoing surgery for colorectal cancer. Therefore, the aim of the present systematic review was to examine the use of commonly employed clinical frailty measures in patients undergoing surgery for colorectal cancer.

## Methods

The protocol for this systematic review was developed using PRISMA-P guidelines, including flowchart [19]. The primary outcome of interest was prevalence of frailty, as defined by measures of frailty, in patients with CRC undergoing surgery. The secondary outcome of interest of this systematic review was the association between frailty and clinical outcomes in those undergoing surgery for CRC. Clinical outcomes recorded where the incidence of post-operative complication (using both Clavien-dindo classification or descriptive definitions), 30-day mortality and overall survival. Patient demographic details, TNM stage, frailty measure used and the prevalence of frailty within the population were all recorded.

A literature search was made of the US National Library of Medicine (MEDLINE) and PubMed, from the start of the relevant database to the 3rd of May 2021. The search terms used were related to the following key words: “frailty”, “colon”, “rectal”. “colorectal”, “cancer”, “elderly”, “surgery”, “resection”, “frailty index”, “frailty score”, “Canadian Study of Health and Aging-Clinical Frailty Scale”, “CSHA-CSF”, “Fried frailty phenotype”, “Onco-geriatric screening tool”, “G8 questionnaire”, “Modified frailty index-5” and “MFI-5”, “Modified frailty index-11”, “MFI-11”, “Edmonton Frail Scale”, and “Groningen Frailty Indicator”. The search terms were chosen following multiple pilot searches using more inclusive terms that returned large numbers of abstracts which on initial assessment were irrelevant to the present review topic.

The title and abstracts of all studies returned by the search were examined for relevance by two researchers (JM and RDD). The full text of each study deemed potentially relevant was obtained and analysed. Review articles, non-English papers, duplicate data sets and abstract only results were excluded. To be included a study had to examine the prevalence of frailty, using any of the common frailty scoring measures as previously described, in patients undergoing surgery for colorectal cancer. Furthermore, the relationship with frailty and post-operative complications, with severity defined by Clavien Dindo classification or descriptive definitions, 30-day mortality or overall survival. Reference lists of included papers, and excluded systematic reviews and meta-analyses, were then hand searched for additional relevant studies. Uncertainties in selection and extraction were resolved by discussion with the senior author (DCM), and the final decision made by the senior author. The Newcastle–Ottawa Scale (NOS) was used to assess the quality of included studies.

Assessment of the risk of bias was carried out using the Risk Of Bias In Non-randomized Studies of Interventions (ROBINS-I) tool [20]. Meta-analysis was not performed because of significant heterogeneity among study methodology, populations and outcomes measured. Ethical approval was not required for the present study as this was a systematic review of published data.

## Results

**Fig. 1:**
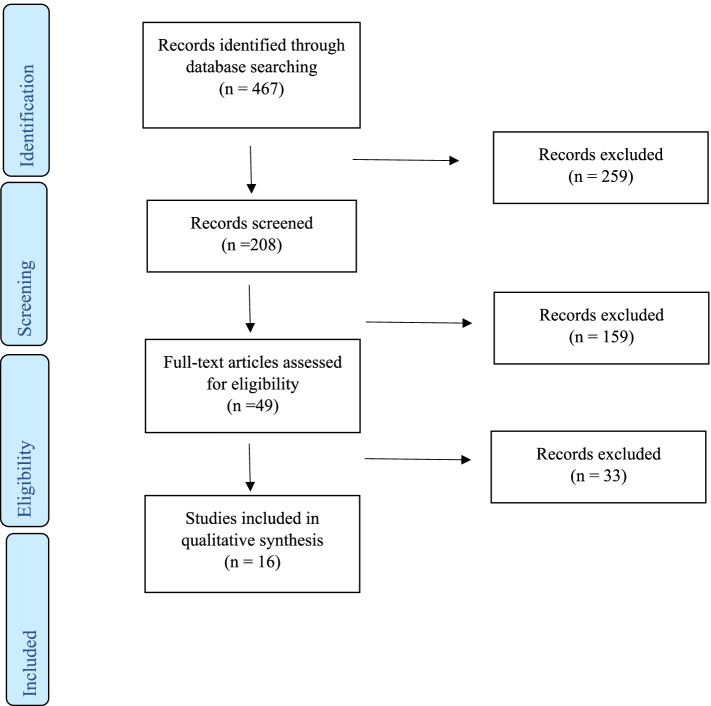
Flow diagram of literature search and included/excluded studies

A total of 467 studies were identified on initial search of the Medline and PubMed databases. Following the exclusion of duplicates by the screening of titles, 208 abstracts were reviewed. 49 full papers were then deemed suitable for review, with 15 meeting inclusion criteria for qualitative analysis. Of 34 studies deemed not to meet the eligibility criteria and therefore excluded, reasons include: post-operative outcome measured other than those listed above (*n* = 13), duplicate publication of the same population (*n* = 4), inclusion of another cancer subtype in the cohort examining the relationship with frailty and post-operative outcomes (*n* = 1), cohort included patients with non-cancerous pathology such as inflammatory bowel disease (*n* = 5), studies in which patients did not undergo surgery or received anti-cancer treatment only (*n* = 9) and lastly, studies that failed to report the prevalence of frailty or threshold used to define frailty in the population (*n* = 2) (See Fig. 1).

### Qualitive Analysis

**Fig. 2:**
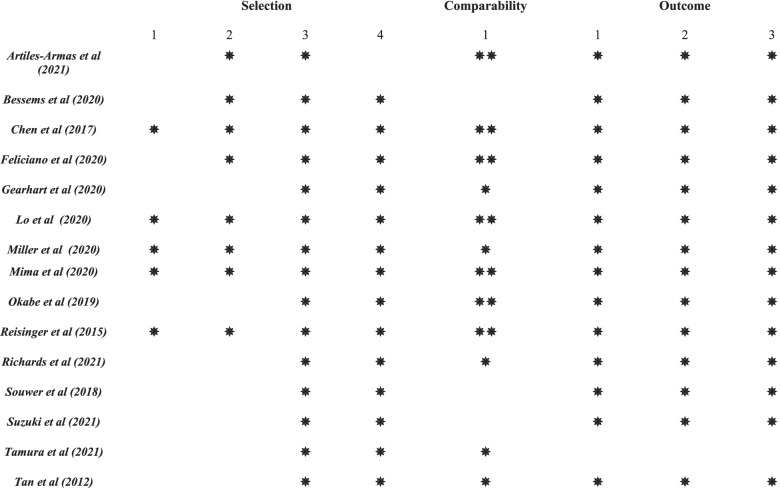
Quality assessment of included studies using the Newcastle–Ottawa Scale (NOS)

Fifteen studies (6 prospective and 9 retrospective, 97, 898 patients) were included in the qualitative analysis (See Table 1). The breakdown of quality of these studies using the Newcastle–Ottawa Scale (NOS) is shown in Fig. 2. To define frailty, three studies used the CSHA-CFS, three used the G8 questionnaire, two used Fried Frailty phenotype and four used the MFI-5 score. The MFI-11, Groningen frailty index and Edmonton frail scale were each used in one study. Of these studies, twelve reported the incidence of post-operative complications, four studies reported the incidence of thirty-day mortality and three studies reported long-term survival outcomes. In all but two studies reporting the median/mean age [21, 22], the majority included patients aged 70 years or older. Over 80% (*n* = 81, 803) of patients included were from a single study by Lo and co-workers [23], who found approximately 20% of patients were frail (MFI-5 ≥ *2).* Tamura and co-workers reported the highest prevalence of frailty at 56% (*n* = 278) in a cohort of 500 patients using the G8 questionnaire [24]. 12% was the lowest prevalence of frailty reported in the included studies, in a study by Chen and co-workers of 1928 patients, that used the MFI-5 index [21].

**Table 1:** Characteristics of included studies

*Study*	*Design*	*Patient (n* =*)*	*Country*	*Frailty screening tool*	*Prevalence of frailty (%)*	*Timing of assessment*	*Age (Median/ Mean; years)*	*% Male/Female*	*TNM Stage*
Artiles-Armas et al. (2021,) [34]	*Prospective*	*149*	*Spain*	*Clinical Frailty Scale*	*42 CSHA CFS* ≥ *4)*	*Pre-operative*	*75*	*64/36*	*I-IV*
Bessems et al. (2021,) [33]	*Retrospective*	*132*	*Netherlands*	*Geriatric 8 questionnaire*	*40 G8* ≤ *14)*	*Pre-operative*	*78*	*58/42*	*I-IV*
Chen et al. (2018,) [21]	*Retrospective*	*1928*	*USA*	*Modified Frailty Index (MFI-5)*	*12* (M*FI* ≥ *2)*	*Pre-operative*	*59*	*55/45*	*IV*
Feliciano et al. (2020,) [22]	*Prospective*	*691*	*USA*	*Fried Frailty phenotype*	*18 (Fried* ≥ *3/5 criteria)*	*Pre-operative*	*63*	*Female only*	*NR*
Gearhart et al. (2020,) [25]	*Retrospective*	*1676*	*USA*	*Modified Frailty Index (MFI-5)*	*25 (MFI* ≥ *2)*	*Pre-operative*	*75*	*50/50*	*NR*
Lo et al. (2020,) [23]	*Retrospective*	*81, 803*	*USA*	*Modified Frailty Index (MFI-5)*	*20 (MFI* ≥ *2)*	*Pre-operative*	*NR (59%* ≥ *65)*	*50/50*	*I-IV*
Miller et al. (2020,) [26]	*Retrospective*	*9,252*	*USA*	*Modified Frailty Index (MFI-5)*	*15 (MFI* ≥ *2)*	*Pre-operative*	*NR (25%* ≥ *65)*	*58/42*	*I-IV*
Mima et al. (2020, [35])	*Retrospective*	*729*	*Japan*	*Clinical Frailty Scale*	*35 (CSHA CFS* ≥ *4)*	*Pre-operative*	*NR (46%* ≥ *75 years)*	*53/47*	*I-III*
Okabe et al. (2019,) [27]	*Prospective*	*269*	*Japan*	*Clinical Frailty Scale*	*29 (CSHA CFS* ≥ *4)*	*Pre-operative*	*80*	*62/38*	*III-IV*
Reisinger et al. (2015,) [28]	*Retrospective*	*310*	*Netherlands*	*Groningen Frailty Indicator*	*25 (GFI* ≥ *5)*	*Pre-operative*	*NR (51%* ≥ *70)*	*50/50*	*II-IV*
Richards et al. (2021,) [29]	*Prospective*	*86*	*New Zealand*	*Edmonton Frailty Scale*	*14 (EFS* ≥ *8)*	*Pre-operative*	*76*	*50/50*	*I-IV*
Souwer et al. (2018,) [30]	*Retrospective*	*139*	*Netherlands*	*Geriatric 8 questionnaire*	*50 (G8* ≤ *14)*	*Pre-operative*	*77.8*	*55/45*	*I-III*
Suzuki et al. (2021,) [31]	*Retrospective*	*151*	*Japan*	*Modified Frailty Index (MFI-11)*	*35 (MFI* ≥ *3)*	*Pre-operative*	*84*	*44/56*	*NR*
Tamura et al. (2021,) [24]	*Prospective*	*500*	*Japan*	*Geriatric 8 questionnaire*	*56 (G8* ≤ *14)*	*Pre-operative*	*76*	*58/42*	*I-IV*
Tan et al. (2012,) [32]	*Prospective*	*83*	*Japan*	*Fried Frailty Phenotype*	*28 (Fried* ≥ *3/5 criteria)*	*Pre-operative*	*81.2*	*NR*	*NR*

### Studies reporting incidence of post-operative complications

The relationship between frailty and post-operative complications is shown in Table 2. Twelve studies including 96,329 patients reported the incidence of post-operative complications in frail patients undergoing surgery for colorectal cancer [21, 23–33]. Post-operative complications included ranged from CD ≥ 1 in three studies, CD ≥ 2 in four studies and CD ≥ 3 in five studies. In one of the three studies reporting the incidence of grade ≥ 1 complications, frailty was significantly associated with the development of post-operative complications on univariate analysis (*p* = 0.038, [33]). Three out of the four studies reporting the incidence of grade ≥ 2 complications, found that frailty was associated with the incidence of post-operative complications [26, 31, 32]. Furthermore, this association remained significant on multivariate binary logistics regression analysis in two studies [26, 32]. Lastly, in studies reporting the incidence of serious complications i.e., grade ≥ 3, three reported that frailty was significantly associated with post-operative complications on multivariate binary logistics regression analysis [21, 23, 27]. Of the studies showing an association with frailty and the incidence of post-operative complications on multivariate analysis (See Table 2), the strength of this was found to be moderate in two studies [21, 23] and strong in the other three [26, 27, 32].

**Table 2:** Studies reporting the relationship between frailty and post-operative complications in patients undergoing surgery for colorectal cancer

*Study*	*Design*	*Patient (n* =*)*	*Country*	*Frailty screening tool*	*Prevalence of frailty (%)*	*Timing of assessment*	*Age (Median/ Mean; years)*	*% Male/Female*	*TNM Stage*	*Clavien-dindo classification of complication*	*Comments*
Bessems et al. (2021,) [33]	*Retrospective*	*132*	*Netherlands*	*Geriatric 8 questionnaire*	*40 (G8* ≤ *14)*	*Pre-operative*	*78*	*58/42*	*I-IV*	*1 or above*	*Frailty associated with complication incidence on UV analysis (P* = *0.038)*
Chen et al. (2018,) [21]	*Retrospective*	*1928*	*USA*	*Modified Frailty Index (MFI-5)*	*12* (M*FI* ≥ *2)*	*Pre-operative*	*59*	*55/45*	*IV*	*3 or above*	*Frailty associated with complication incidence on MV binary log regression (OR 2.12, 95% CI 1.47–3.04, P* < *0.001)*
Gearhart et al. (2020,) [25]	*Retrospective*	*1676*	*USA*	*Modified Frailty Index (MFI-5)*	*25 (MFI* ≥ *2)*	*Pre-operative*	*75*	*50/50*	*NR*	*2 or above*	*Frailty not associated with complication incidence on MV binary log regression (P* = *0.19)*
Lo et al. (2020,) [23]	*Retrospective*	*81, 803*	*USA*	*Modified Frailty Index (MFI-5)*	*20 (MFI* ≥ *2)*	*Pre-operative*	*NR (59%* ≥ *65)*	*50/50*	*I-IV*	*3 or above*	*Frailty associated with complication incidence on MV binary log regression (OR 1.56, 95% CI 1.07–2.25, P* = *0.018)*
Miller at sl (2020,) [26]	*Retrospective*	*9,252*	*USA*	*Modified Frailty Index (MFI-5)*	*15 (MFI* ≥ *2)*	*Pre-operative*	*NR (25%* ≥ *65)*	*58/42*	*I-IV*	*2 or above*	*Frailty associated with complication incidence on MV binary log regression (OR 6.7, 95% CI 4.5–10.0, P* < *0.001)*
Okabe et al. (2019,) [27]	*Prospective*	*269*	*Japan*	*Clinical Frailty Scale*	*29 (CSHA CFS* ≥ *4)*	*Pre-operative*	*80*	*62/38*	*III-IV*	*3 or above*	*Frailty associated with complication incidence on MV binary log regression OR 3.42, 95% CI 1.62–7.29. P* = *0.001)*
Reisinger et al. (2015,) [28]	*Retrospective*	*310*	*Netherlands*	*Groningen Frailty Indicator*	*25 (GFI* ≥ *5)*	*Pre-operative*	*NR (51%* ≥ *70)*	*50/50*	*II-IV*	*3 or above*	*Frailty not associated with complication incidence on UV binary log regression (P* = *0.19)*
Richards et al. (2021,) [29]	*Prospective*	*86*	*New Zealand*	*Edmonton Frailty Scale*	*14 (EFS* ≥ *8)*	*Pre-operative*	*76*	*50/50*	*I-IV*	*3 or above*	*Frailty not associated with complication incidence on MV binary log regression P* = *0.62)*
Souwer et al. (2018,) [30]	*Retrospective*	*139*	*Netherlands*	*Geriatric 8 questionnaire*	*50 (G8* ≤ *14)*	*Pre-operative*	*77.8*	*55/45*	*I-III*	*1 or above*	*Frailty not associated with complication incidence on UV analysis (P* = *0.7)*
Suzuki et al. (2021,) [31]	*Retrospective*	*151*	*Japan*	*Modified Frailty Index (MFI-11)*	*35 (MFI* ≥ *3)*	*Pre-operative*	*84*	*44/56*	*NR*	*2 or above*	*Frailty associated with complication incidence on UV analysis (P* = *0.02)*
Tamura et al. (2021,) [24]	*Prospective*	*500*	*Japan*	*Geriatric 8 questionnaire*	*56 (G8* ≤ *14)*	*Pre-operative*	*76*	*58/42*	*I-IV*	*1 or above*	*Frailty not associated with complication incidence on UV binary log regression (P* = *0.355)*
Tan et al. (2012,) [32]	*Prospective*	*83*	*Japan*	*Fried Frailty Phenotype*	*28 (Fried* ≥ *3/5 criteria)*	*Pre-operative*	*81.2*	*NR*	*NR*	*2 or above*	*Frailty associated with complication incidence on MV binary log regression (OR 4.08, 95% CI, 1.43–11.6, P* = *0.006)*

*UV* Univariate*, MV* Multivariate*, OR* Odds Ratio

### Studies reporting incidence of thirty-day mortality

The relationship between frailty and thirty-day mortality is shown in Table 3. Four studies including 9,880 patients reported the incidence of thirty-day mortality in frail patients undergoing surgery for colorectal cancer [26, 28, 30, 34]. Two studies, one using the CSHA-CFS [34] and the other using the MFI-5 score [26], reported that frailty was significantly associated with thirty-day mortality. In the latter, this association remained significant on multivariate binary logistics regression analysis (*p* < 0.001, [26]. The strength of the association was found to be strong *(OR 20.8, 95% CI 6.2–70.0, P* < *0.001, See* Table 2*).* In the remaining two studies, the association was not significant on univariate analysis [28, 30].

**Table 3:** Studies reporting the relationship between frailty and thirty-day mortality patients undergoing surgery for colorectal cancer

*Study*	*Design*	*Patient (n* =*)*	*Country*	*Frailty screening tool*	*Frailty Prevalence*	*Timing of assessment*	*Age (Median/ Mean; years)*	*% Male/Female*	*TNM Stage*	*Comments*
Artiles-Armas et al. (2021,) [34]	*Prospective*	*149*	*Spain*	*Clinical Frailty Scale*	*42 (CSHA CFS* ≥ *4)*	*Pre-operative*	*75*	*64/36*	*I-IV*	*Frailty associated with increased mortality on UV analysis* *(P* = *0.009)*
Miller et al. (2020,) [26]	*Retrospective*	*9,252*	*USA*	*Modified Frailty Index (MFI-5)*	*15 (MFI* ≥ *2)*	*Pre-operative*	*NR (25%* ≥ *65)*	*58/42*	*I-IV*	*Frailty associated with increased mortality on MV binary log regression* *(OR 20.8, 95% CI 6.2–70.0, P* < *0.001)*
Reisinger et al. (2015,) [28]	*Retrospective*	*340*	*Netherlands*	*Groningen Frailty Indicator*	*25 (GFI* ≥ *5)*	*Pre-operative*	*69*	*50/50*	*II-IV*	*Frailty not associated with increased mortality on UV binary log regression* *(P* = *0.72)*
Souwer et al. (2018,) [30]	*Retrospective*	*139*	*Netherlands*	*Geriatric 8 questionnaire*	*50 (G8* ≤ *14)*	*Pre-operative*	*77.8*	*55/45*	*I-III*	*Frailty not associated with increased mortality on UV binary log regression* *(P* = *1.00)*

*UV* Univariate, *MV* Multivariate*, OR* Odds Ratio

### Studies reporting overall survival

The relationship between frailty and overall survival is shown in Table 4. Three studies including 1, 569 patients reported the association between frailty and overall survival [22, 34, 35]. Artiles-Armas and co-workers reported a mean follow-up of 5 years only [34]. Mima and co-workers reported a median follow-up of 3.5 years (interquartile range: 2.5–5.1 years, [35]. Feliciano and co-workers reported a median follow-up of 5.8 years (interquartile range: 1 month-19.9 years, [22]. Frailty, defined by the CSHA-CFS and frailty phenotype, was found to be significantly associated with overall survival in two studies (Both, *P* < 0.001 [22, 35]. In both studies this association was found to be of moderate strength *(HR 2.40, 95% CI 1.40–2.99, P* < *0.001 and HR 1.94, 95% CI 1.39–2.69, P* < *0.001, See* Table 4*).*

**Table 4:** The relationship between frailty and overall survival

*Study*	*Design*	*Patient (n* =*)*	*Country*	*Frailty screening tool*	*Frailty Prevalence*	*Timing of assessment*	*Age (Median/ Mean; years)*	*% Male/Female*	*TNM Stage*	*Survival Outcome Measured*	*Mean/Median Follow-up (Years)*	*Comments*
Artiles-Armas et al. (2021) [34]	*Prospective*	*149*	*Spain*	*Clinical Frailty Scale*	*42 (CSHA CFS* ≥ *4)*	*Pre-operative*	*75*	*64/36*	*I-IV*	*Overall survival*	*5*	*Frailty not associated with reduced survival on UV binary log regression (P* = *0.249)*
Feliciano et al. (2020) [22]	*Prospective*	*691*	*USA*	*Frailty phenotype*	*18 (Fried* ≥ *3/5 criteria)*	*Pre-operative*	*63*	*Female only*	*NR*	*Overall survival*	*5.8*	*Frailty associated with OS on MV binary log regression (HR 1.94, 95% CI 1.39–2.69, P* < *0.001)*
Mima et al. (2020) [35]	*Retrospective*	*729*	*Japan*	*Clinical Frailty Scale*	*35 (CSHA CFS* ≥ *4)*	*Pre-operative*	*NR (46%* ≥ *75 years)*	*53/47*	*I-III*	*Overall survival*	*3.5*	*Frailty associated with OS on MV binary log regression (HR 2.40, 95% CI 1.40–2.99, P* < *0.001)*

*UV* Univariate, *MV* Multivariate, *HR* Hazard ratio

### Assessment of bias

The ROBINS-I tool was used to assess the risk of bias in included studies. All fifteen of the included studies were deemed at moderate or severe risk of bias overall. Bias due to confounding factors, selection bias and reporting of results was prevalent.

## Discussion

To our knowledge, the present systematic review examining the relationship between frailty and post-operative outcomes in older adults undergoing surgery for CRC is the most comprehensive to date, including 15 studies totalling 97, 898 patients. The results show that frailty is common in older adults undergoing surgery for CRC and would appear to be moderately and negatively associated with clinical outcomes including the incidence of post-operative complications, 30-day mortality and overall survival. However, due to the limited literature it is still not at present clear which frailty screening measures have clinical utility in the treatment of CRC. Furthermore, the basis of the relationship between frailty and post-operative outcomes is unclear.

Frailty is a spectrum that reflects the systemic, global burden of human aging and erosion of the patients homeostatic reserve [36]. As such one would expect that frailty would be associated with both short- and long-term adverse outcomes. This is in keeping with a recent review by Fagard and co-workers, that included four prospective studies totalling 486 patients, who found that frail patients with CRC were more at risk of adverse outcomes following surgery [37]. However, frailty was only found to be adversely associated with clinical outcomes in 9 of the 15 studies included. The results raise doubts on the reliability of observations in some of the included studies and the clinical utility of certain frailty measures. This highlights the need for frailty screening measures that assess a broad range of domains but are simple and time-efficient enough to be readily employed in clinical practice. Potential examples are the MFI-5 shown to have prognostic value in older adults undergoing surgery for CRC [38, 39] and the CSHA-CFS which is quick to perform, requires limited training of staff and has been shown to have good inter-observer reliability [40, 41].

Frailty is of growing interest and importance across different subspecialities of medicine. It is thought to encompass not only age, but a number of recognised domains including functional status, malnutrition, co-morbidity, cognition, socio-economic and psychological factors [42, 43]. Recent work by Miller and co-workers reported that frailty, but not age, had an independent prognostic value in patients with colorectal cancer [26]. Furthermore, of the seven frailty screening measures included in the present review, only the G8 questionnaire included the assessment of age [44]. The results suggest that simply assessing older adults is insufficient and that those who are functionally restricted, co-morbid or cachexic are likely to also be frail. Indeed, frailty been associated with pre-operative host factors including malnutrition, sarcopenia and inflammation [45]. However, these factors are all independently associated with adverse clinical outcomes in patients undergoing surgery for CRC. Therefore, it remains unclear if frailty per se has independent prognostic value or is simply reflective of the functional and nutritional reserve of the patient to the stress of surgery. Against this background it is of interest that many of the innovations in surgery and anaesthesia in recent decades have been directed at minimising the stressors on the physiological reserve [46]. Indeed, robot assisted surgery has been reported to be associated with better clinical outcomes in older adults with CRC [47, 48].

Frailty and sarcopenia are prevalent and important determinants of functional status and independence in older adults [49, 50]. Indeed, both have been shown to have prognostic value in patients undergoing surgery for colorectal cancer [51, 52]. However, while there is overlap between the conditions [53], the terms are not synonymous. Specifically, sarcopenia is one of many causes of functional impairment- the hallmark of frailty [54]. Therefore, while frailty and sarcopenia may exist independently, whether frailty has independent prognostic value in patients with colorectal cancer is unclear. Further research is required to delineate the relationship between frailty and clinical outcomes, in non-sarcopenic older adults undergoing surgery for colorectal cancer.

Malnutrition, like sarcopenia, is another recognised prognostic factor in those with cancer [55], shown to be prevalent in elderly, frail patients [56, 57]. However, the relationship between malnutrition, muscle mass and functional status in frail patients is poorly understood. Much of the present literature relating to therapeutic interventions in frailty comprises of studies attempting to optimize skeletal muscle mass, with physical activity and nutritional supplementation, to optimize functional status [58–60]. Work by Tieland et al. found that dietary protein supplementation improved physical performance in frail patients, but skeletal muscle mass was not increased [61]. Furthermore, work by Bessems et al. demonstrated that frailty, screened using the G8 questionnaire in addition to 4-m gait speed test, was associated with the incidence of post-operative outcomes in a cohort where malnutrition was prevalent [33]. However, the results contrast those of another similar cohort size study from the Netherlands that found the G8 questionnaire had no prognostic value in patients undergoing surgery for colorectal cancer [30]. The disparity between the results of studies suggest that further studies will be required to tease out the relationship between malnutrition, sarcopenia, and functional status in frail patients with cancer.

Inflammation is recognized as one of the seven pillars of aging [62]. A low grade, chronic systemic inflammatory state is observed with advancing age [63]. Recent systematic reviews have shown that frailty is associated with elevated systemic inflammatory markers including CRP and IL-6 [64]. Although, the pathophysiological changes underlying and preceding frailty are not clearly understood, it is plausible that an exaggerated systemic inflammatory response is responsible [64]. Furthermore, systemic inflammation is associated with other recognised domains of frailty including malnutrition [65], sarcopenia and fatigue [66], commonly found in patients with advanced cancer. Therefore, the success of therapeutic interventions to arrest or reverse frailty may require modulation of the systemic inflammatory response, in addition to nutritional supplement and physical exercise [67], as proposed for the pre-habilitation of patients with advanced cancer [68].

There are several limitations of the present systematic review. Firstly, the studies included were mainly retrospective and are therefore subject to confounding factors and selection bias. An example being that patients who were deemed to be frail at diagnosis are more likely to undergo minimally invasive laparoscopic surgery, associated with better outcomes in colorectal cancer [46]. Furthermore, those who were deemed to be very frail are unlikely to be considered for surgery and be palliated. Secondly, the absence of a meta-analysis or a pooled prevalence. Neither were considered to be appropriate because of significant heterogeneity of the studies and the large number of observations confined to a few individual studies. Lastly, the majority of studies included in the review were of patients who underwent resection of CRC with curative intent. Therefore, future studies will be required to assess the prevalence and prognostic value of frailty in those with advanced disease.

In conclusion, frailty was common in older adults undergoing surgery for colorectal cancer, across a range of frailty screening measures. Which of these has the greatest utility in clinical practice is unclear and requires further study. Furthermore, while frailty would appear to be moderately associated with post-operative outcomes, the basis of this relationship also remains unclear. Specifically, if frailty per se has an independent prognostic value or is simply reflective of the nutritional and functional reserve of the patient.

## Data Availability

Raw data will be made available on request to the senior author (DCM).
